# Whey as a Diet in Typhoid Fever

**Published:** 1901-11-09

**Authors:** 


### WHEY AS A DIET IN TYPHOID FEVER.

One, if not the great, objection to the use of milk as a diet in typhoid fever arises from the large amount of curd which it produces. This, in its passage through the intestines, often decomposes and gives rise to gaseous distention, besides forming in itself a hard and injurious form of excrement. The question then arises what to give in place of milk? To this various answers have been given, and of late years a strong tendency has shown itself to give typhoid patients a far more varied dietary than was generally permitted a few years ago. No one can doubt, however, that there are great advantages in the use of a standard diet, and that under ordinary circumstances, that is when the medical attendant cannot be almost constantly on the spot, there are many dangers in allowing any latitude in this matter. In hospitals where an intelligent sister is over the nurses in each ward the matter is simplified a good deal, but even there, with a ward full of patients taking all sorts of fancy diets, danger would be by no means entirely eliminated. A large number of cases of fever, however, are, and probably for long must be, nursed at home, and nursed often by women who, great as may be their goodwill, can hardly be trusted outside the definite lines of a fixed and simple dietary. Under these circumstances Dr. Prideaux Selby[1] has done well to remind us of the great advantages of whey as a diet in typhoid fever. Comparing the composition of milk and of whey, he shows that the latter contains about half as much solids as the former. The loss in fat is 2·2 per cent., and in albumin 2·5 per cent. Whey, therefore, practically consists of a solution of milk sugar, with a small amount of fat, albumin, and salts. Clearly a diet consisting of say four pints of whey per diem is starvation if one compares it with the generally accepted physiological requirements of a male adult at rest, and, as Dr. Selby says, it is a remarkable thing that such good results should have been obtained with a dietary so apparently deficient. It would have been a matter of much interest if he had added to his table of results, a column showing the loss of weight during the treatment. On going through this table we note that in a not inconsiderable number of the cases either milk, or chicken broth, or beef tea was given in addition to the whey at some time or other of the fever, and also that more than two-thirds of the cases received alcohol. There were, however, only two deaths among the 75 cases recorded, and the question really does come before us with much force whether it may not be better to keep a patient well within the physiological requirements in regard to food, leaving him to feed to a large extent upon himself and "keeping him going," if one may so say, by the use of a little stimulant, than to give him a diet which, although containing a much nearer approximation to what he requires according to physiological standards, will undoubtedly be only in part digested, and may probably by its indigestion lead to serious complications. The whey is prepared by adding a sufficiency of rennet (the quantity, we imagine, must vary with the preparation used) to the milk and warming it slowly in a pan until it curdles. This takes about 20 minutes. Then the curd is broken up and the whole is strained through fine muslin. A quart of milk yields about six ounces of curd. More cream can be added to the whey if thought desirable. The quantity given varies from a pint and a-half to six pints. It is not necessary to limit the quantity.

[1] Lancet, Nov. 2.

